# Bacterial zinc uptake regulator proteins and their regulons

**DOI:** 10.1042/BST20170228

**Published:** 2018-07-31

**Authors:** Alevtina Mikhaylina, Amira Z. Ksibe, David J. Scanlan, Claudia A. Blindauer

**Affiliations:** 1 Department of Chemistry, University of Warwick, Coventry CV4 7AL, U.K.; 2 School of Life Sciences, University of Warwick, Coventry CV4 7AL, U.K.

**Keywords:** bacteria, metal ions, zinc-responsive transcription factors, zinc uptake regulator, Zur

## Abstract

All organisms must regulate the cellular uptake, efflux, and intracellular trafficking of essential elements, including d-block metal ions. In bacteria, such regulation is achieved by the action of metal-responsive transcriptional regulators. Among several families of zinc-responsive transcription factors, the ‘zinc uptake regulator’ Zur is the most widespread. Zur normally represses transcription in its zinc-bound form, in which DNA-binding affinity is enhanced allosterically. Experimental and bioinformatic searches for Zur-regulated genes have revealed that in many cases, Zur proteins govern zinc homeostasis in a much more profound way than merely through the expression of uptake systems. Zur regulons also comprise biosynthetic clusters for metallophore synthesis, ribosomal proteins, enzymes, and virulence factors. In recognition of the importance of zinc homeostasis at the host–pathogen interface, studying Zur regulons of pathogenic bacteria is a particularly active current research area.

## Introduction

For a long time, there has been a prevailing assumption that zinc is not a particularly important element for prokaryotes. Indeed, as recently as 2010, the contention that ‘the akaryotic superkingdoms of Archaea and Bacteria eschew Zn’ was highlighted as a finding of a phylogenomic analysis of protein structures [1]. It is certainly true that the proportion of zinc-requiring proteins in prokaryotes (5–6%) is significantly lower than that in eukaryotes (9–10%) [2], but this should not detract us from the fact that zinc is also essential for bacteria, and that bacterial zinc homeostasis plays rather critical roles in a variety of contexts. Much recent attention has focused on pathogenic bacteria [3], where both extremely efficient zinc acquisition and enhanced zinc tolerance contribute to survival in the host, virulence, and overall pathogenicity. In zinc-poor environments, bacterial cells are capable of concentrating zinc by several thousand-fold [4]; such highly efficient zinc acquisition is critical in the face of the host's immune response where the availability of zinc, iron, and manganese is severely restricted [5,6], a phenomenon referred to as ‘nutritional immunity’ [7]. Conversely, high zinc concentrations are part of the toxic cocktail that macrophages use to kill pathogens encapsulated in their phagosomes [8] — but not always successfully. Zinc toxicity is thought to be at least partially due to mis-metallation of other metalloproteins, as Zn^2+^ forms more stable complexes than most other essential metal ions [9], so intracellular concentrations must be stringently regulated.

**Figure 1.**
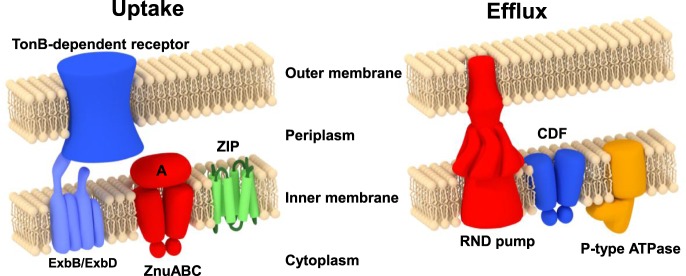
Overview of the major players in bacterial zinc uptake and efflux, illustrated for a Gram-negative bacterium. Proteins for import include members of the ZIP (zinc-iron permease) family and members of the ATP-binding cassette (ABC) superfamily. The latter systems consist of a membrane-bound permease, an ATPase, and a protein that is periplasmic in Gram-negative bacteria or on the cell surface in Gram-positive bacteria. These systems are usually named ZnuABC (Gram-negative bacteria) or AdcABC (Gram-positive bacteria), although this distinction is not consistently adhered to. A third label used frequently for such zinc importers is TroABC. Exporters include P-type ATPases, members of the cation–diffusion facilitator (CDF) family, and tripartite RND (root–nodulation–cell division) systems [3,10]. Regulatory proteins and further processes are explained in the main text.

Zinc homeostasis in bacteria is maintained by means of a few critical processes, including zinc import and export (Figure 1), intracellular zinc binding, and zinc-sensing [3,10,11]. The fluxes of Zn^2+^ in and out of the cell are, in the first instance, controlled by the levels at which importer and exporter proteins are present. In bacteria, these levels are to a large extent controlled at the transcriptional level [12], with available intracellular Zn^2+^ being sensed by several types of zinc-responsive transcription factors [13]. These include members of the ferric uptake regulator (Fur) family (Zur; [4,14–17]) and the MarR/SlyA family (AdcR) [18–20] to up-regulate zinc import, and members of the MerR (ZntR) and ArsR/SmtB families (SmtB, ZiaR, and CzrA; [21–24]), to increase zinc export and/or intracellular sequestration. Besides Zur, the Fur family (COG0735) [25,26] also includes Fur [27–29], Nur [30,31], and Mur [32,33], which sense other metal cations (Fe^2+^, Ni^2+^, and Mn^2+^, respectively). Further members are PerR [34,35] and Irr [36], which sense cytoplasmic peroxide and haem, respectively.

**Figure 2.**
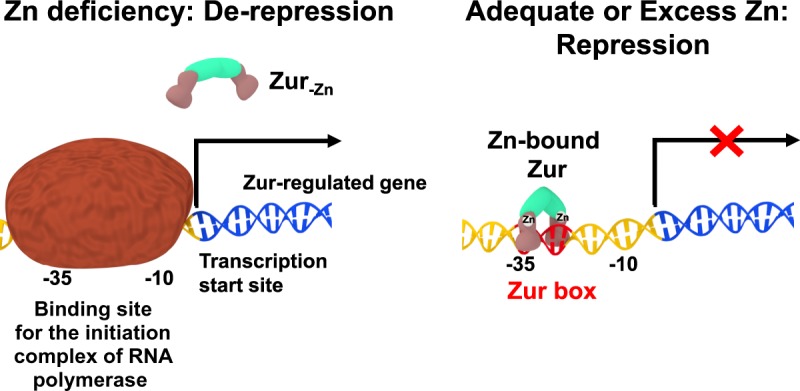
Schematic illustration of canonical regulation of transcription by Zur. Zinc-bound Zur (right-hand panel; see below for further details) represses transcription by binding to specific DNA sequences (Zur boxes) in the promoter region of Zur-regulated genes and thus inhibits initiation of transcription. When cells are deprived of zinc, demetallated Zur has a dramatically reduced affinity for DNA, allowing transcription to occur.

In principle, zinc-sensing transcription factors can work as either repressors or activators. The unifying point is that in each case Zn^2+^ binding alters the affinity to the cognate DNA. This is achieved by allostery; the bound Zn^2+^ alters the conformation and/or the structural dynamics of the protein, which can either enhance or decrease the affinity for specific DNA sequences [37]. Like other Fur family proteins, Zur proteins act predominantly as transcriptional repressors when Zn^2+^ is bound, by blocking the binding site for the RNA polymerase transcription initiation complex (Figure 2). Often, the Zur-specific DNA sequences (Zur boxes) overlap with the −35 position in the promoter regions of regulated genes. This review examines structures and properties of Zur proteins and their typical mode of action, and gives a comprehensive overview of which genes are Zur-regulated. This includes the recent discovery that Zur proteins may also act as transcriptional activators.

## Structures and mode of action of Zur proteins

**Figure 3.**
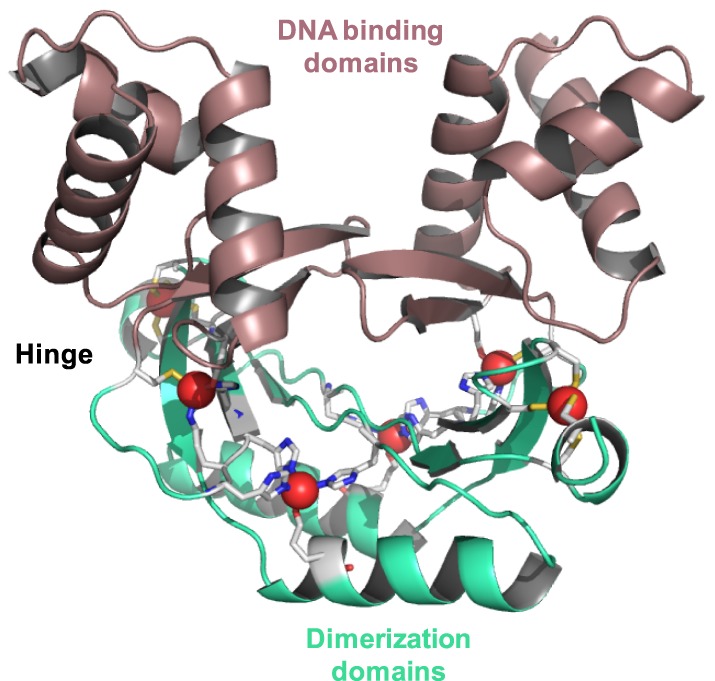
X-ray crystal structure of dimeric *S. coelicolor* Zur (pdb 3mwm) [38]. DNA-binding domains are shown in maroon and dimerisation domains in green. Zinc ions are shown as red spheres, nitrogen atoms in blue, sulfur in yellow, and carbon in light grey.

Among many 3D structures for Fur family proteins [26], the structures of three Zur proteins have been determined by X-ray crystallography, namely those of *Mycobacterium tuberculosis* FurB (MtZur) [15], *Streptomyces coelicolor* (ScZur; Figure 3) [38], and *Escherichia coli* (EcZur) [17]. All three proteins are homodimeric in solution (Table 1) and at least dimeric in the crystal. Each monomer has two domains: an N-terminal DNA-binding domain that is predominantly α-helical and a C-terminal dimerisation domain containing a three-stranded β-sheet. Its longest β-strand pairs up with its counterpart in the dimer. The two domains in each monomer are connected by a hinge.

**Table 1:** Summarised data for expression constructs and purification protocols, oligomerisation states, and Zn stoichiometry of Zur proteins from different organisms

Organism	Expression and purification	Oligomerisation	Stoichiometry (Zn per monomer)	Ref.
*Mycobacterium tuberculosis*	GST-fusion; glutathione sepharose 4B resin	n.d.; assumed dimeric	1.8 ± 0.2 (Bradford assay and FAAS)	[39]
*Mycobacterium tuberculosis*	His-tagged; Ni-IMAC, TEV-cleavage, SEC	Dimer: X-ray crystallography	3 (X-ray crystallography) 1 (post EDTA treatment; μPIXE)	[15]
*Streptomyces coelicolor*	His-tagged; Ni-charged Chelex-100, elution with imidazole, dialysis against 5 mM EDTA	Dimer (analytical ultracentrifugation). No indication of self-association.	n.d.	[16]
*Streptomyces coelicolor*	No tag; Ni-charged NTA column; elution with imidazole gradient; dialysis; no EDTA during purification	Dimer (X-ray crystallography and SEC) for WT, D, and M site mutants. C90S mutant monomeric	3 (X-ray crystallography) 2.4 (WT; ICP-OES) 1.2 (EDTA treated) 1.5 (H84A mutant; D site) 1.0 (C79S mutant; M site) 0.25 (C90S mutant; C site)	[38]
*Bacillus subtilis*	Cell lysis in presence of 2 mM EDTA and 2 mM DTT; heparin affinity, SEC, AEX, dialysis	Dimer (SEC)	0.5–0.8 initially bound; additional 1.4 Zn by titration, giving approximately 2 Zn per monomer ([P] by *A*_277_, [M] by PAR assay and titration with FluoZin3)	[40,41]
*Streptococcus suis*	His- or GST-tag. Ni-IMAC or glutathione Sepharose 4B (PBS). GST-tag removed	Dimer (chemical cross-linking assays)	n.d.	[42]
*Escherichia coli*	Tag-less expression. Lysis in the presence of 6 M urea, and 100 mM DTT, then refolded in presence of 100 μM Zn^2+^. AEX, HIC and SEC	n.d., assumed dimeric	1.4–1.8 (standardised Bradford assay and ICP-AES)	[43]
*Escherichia coli*	Similar to [43]	Dimer (X-ray crystallography) C103S mutant monomeric	2 (X-ray crystallography) 2.8 (WT; ICP-MS) 0.7 (EDTA-treated WT) 2.1 (C103S mutant) 0.0 (EDTA-treated C103S mutant) 2.5 (C88S mutant) 0.6 (EDTA-treated C88S mutant)	[17]
*Salmonella enterica*	His-tagged. Lysis: 1 mM EDTA IMAC, SEC, heparin affinity	n.d., assumed dimeric	Ca. 1 (ICP-MS) Capacity to bind up to two more per monomer	[44,45]
*Paracoccus denitrificans*	MBP fusion tag. Lysis: 1 mM EDTA. Amylose resin column. Tag cleaved by Factor Xa protease, AEX. Apo-Zur by dialysis	Dimer (SEC)	Ca. 1 (ICP-OES), capacity to bind one more Zn per monomer	[46]
*Synechocystis* sp. *PCC6803*	Tag-less expression. Lysis: 100 mM NaCl, 5 mM DTT, 1 mM EDTA, heparin affinity and SEC	n.d., assumed dimeric	1.02 ± 0.15 (ICP-MS) Capacity to bind at least one more Zn per monomer	[47]
*Anabaena* sp*. PCC 7120*	His-tagged. Cell lysis in presence of 2^ ^M GdnHCl (pH 8). Zn-IMAC, dialysis pH 5.5	Mostly monomer, some dimer (SDS–PAGE, denaturing conditions)	1 (ICP-MS) Up to two more binding sites per monomer	[48]

Abbreviations: AEX: anion-exchange chromatography; DTT: dithiothreitol; EDTA: ethylenediaminetetraacetic acid; FAAS: flame atomic absorption spectroscopy; GdnHCl: guanidinium hydrochloride; GST: Glutathione *S*-transferase; HIC: hydrophobic interaction chromatography; His-tag: polyhistidine-tag; ICP-AES/ICP-OES: inductively coupled plasma atomic/optical emission spectroscopy; ICP-MS: inductively coupled plasma mass spectrometry; IMAC: immobilised metal ion affinity chromatography; MBP: maltose-binding protein; n.d: not determined; μPIXE: micro-proton-induced X-ray emission; PAR: pyridyl-azo-resorcinol; SDS–PAGE: sodium dodecyl sulfate–polyacrylamide gel electrophoresis; SEC: size-exclusion chromatography; TEV: tobacco etch virus; WT: wild type.

**Figure 4.**
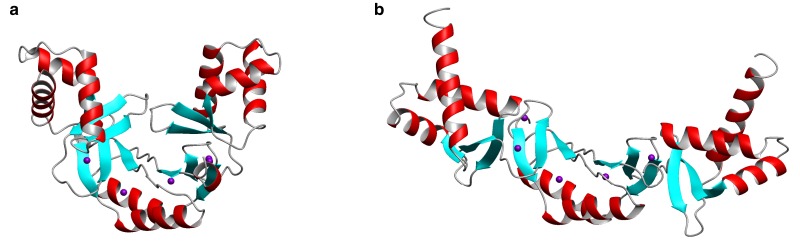
Conformational flexibility of Zur proteins. The “closed” vs. “open” conformation of dimeric Zurs is illustrated by the X-ray structures of (**A**) ScZur (pdb 3mwm) and (**B**) MtZur (pdb 2o03).

Like other members of the Fur family, Zur dimers can adopt at least two conformations, a ‘closed’ one with high DNA-binding affinity and an ‘open’ one with a low DNA-binding affinity (Figure 4). It has been suggested that the DNA-binding domains in Fur-family transcription factors are highly mobile in solution [49,50], with the structures found in single crystals representing ‘frozen-out’ states from an entire range of conformations. Metal binding shifts the conformational equilibrium towards the closed, high-affinity conformation. In the case of Zur, this provides the basis for the allosteric sensing of Zn^2+^.

**Figure 5.**
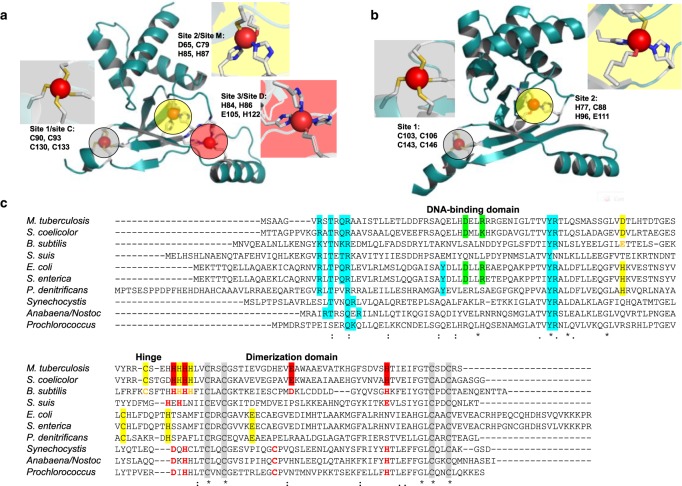
Structural and sensory zinc sites on Zur proteins. (**A**) ScZur (pdb 3mwm [38]) and (**B**) EcZur (pdb 4mtd [17]). The structural sites are highlighted in grey, the single or major sensory site in yellow, and the additional site in ScZur is highlighted in red. (**C**) Sequence alignment of Zur proteins from a variety of species. Residues confirmed to participate in zinc binding by X-ray crystallography are highlighted by red, yellow, and grey backgrounds. Residues involved in DNA binding are highlighted in cyan. Predicted metal-binding residues or sensory sites in Zur proteins that have not been structurally characterised are printed in red or yellow. The two residues forming a salt bridge in EcZur (see the text) are highlighted in green; they are (semi-)conserved in Zur from *Salmonella*, *M. tuberculosis*, and *S. coelicolor*.

This relatively simple picture is somewhat complicated by the fact that each Zur monomer can bind more than one zinc ion (either two or three). EcZur harbours only two Zn sites per monomer [17], while MtZur [15] and ScZur [38] both contain three sites per monomer (see the next section for stoichiometric data for Zurs from other species). In all cases, the Zn^2+^ ions are tetrahedrally co-ordinated (Figure 5).

Not all Zn sites are involved in zinc sensing, the Cys_4_ site 1 (also termed site C; Figure 5A,B) that all three proteins have in common is a non-labile structural site. It is located entirely in the dimerisation domain and stabilises the protein fold by tethering the C-terminus to the β-sheet. Analogous structural Zn sites are also present in a range of other Fur-family proteins that sense other metals [26], and the four Cys residues are also well conserved in Zur proteins that have not yet been structurally characterised (Figure 5C). The single sensory site in EcZur is composed of a mixture of N, S, and O ligands and is derived from residues from both domains and the hinge region, namely a His and a Cys residue from the DNA-binding domain, His77 situated in the hinge region, and a Glu from a short β-strand in the dimerisation domain. Site 2 in MtZur and ScZur sits in an analogous position to this site and also has an N_2_OS composition, even though none of the zinc-coordinating residues is strictly conserved between EcZur and the actinobacterial Zurs (Figure 5C). Their location between the two domains strongly suggests that Zn binding to these sites affects the mutual orientation of the domains, by essentially providing a relatively rigid cross-link through the four bonds between amino acid side chains and Zn^2+^. This cross-link thus favours the prevalence of the ‘closed’ conformation of the dimer.

Thus, site 2 is thought to be the major Zn sensory site in MtZur and ScZur, while the significance of the third Zn site, which is entirely located within the dimerisation domain, is less clear. This site is composed of three His and one Glu residues and was only partially occupied in the MtZur structure [15]. For ScZur, a regulatory role of this site has been confirmed [38]; the case of MtZur is unresolved. The residues forming this site are also (semi-)conserved *in Bacillus subtilis* Zur, but based on metal : protein stoichiometry data, it has been concluded that although the residues are important for dimerisation, they do not bind zinc under physiological conditions [40]. Perplexingly, the position of this site within the protein is very similar to those of the sensory sites in other Fur family proteins. Sequence analysis and structural modelling of cyanobacterial Zur proteins predicted two sites, i.e. the structural site 1, and a sensory site, again with N_2_OS coordination [51]; the predicted residues are also highlighted in Figure 5C.

**Figure 6.**
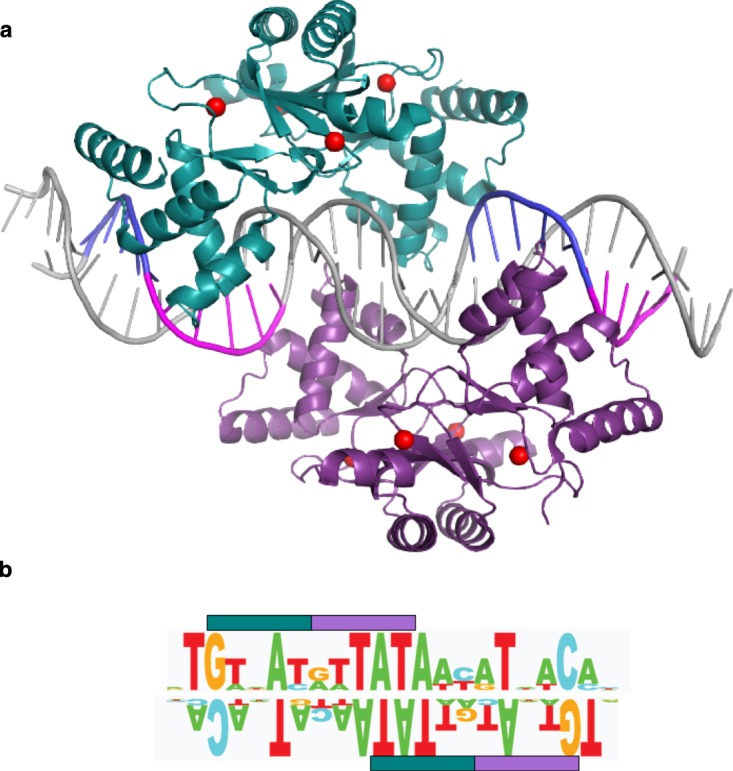
DNA binding by EcZur. (**A**) EcZur in complex with DNA (31 base pairs from the *znuABC* promoter; pdb 4mtd [17]). The two dimers binding to the complete Zur box are shown in green and purple. DNA backbone and bases are shown schematically, with the regions forming interactions with the protein highlighted in blue and magenta. The position of zinc ions is indicated in red. *E. coli* Zur boxes can bind one or two dimers; for the *znuABC* promoter, there is a high degree of cooperativity, leading to the overwhelming prevalence of the complex involving two dimers. (**B**) Illustration of the consensus sequence for the *E. coli* Zur box, with RNNNY (R = purine; Y = pyrimidine; N = any base) motifs important for Zur–DNA interactions highlighted. Each of the bars corresponds to the interaction motif for one monomer. The sequence logo for the consensus sequence is taken from ref. [17].

EcZur is the only Zur protein which has been crystallised in complex with its cognate DNA (Figure 6); this complex has provided insights about which residues are critical for this interaction.

**Figure 7.**
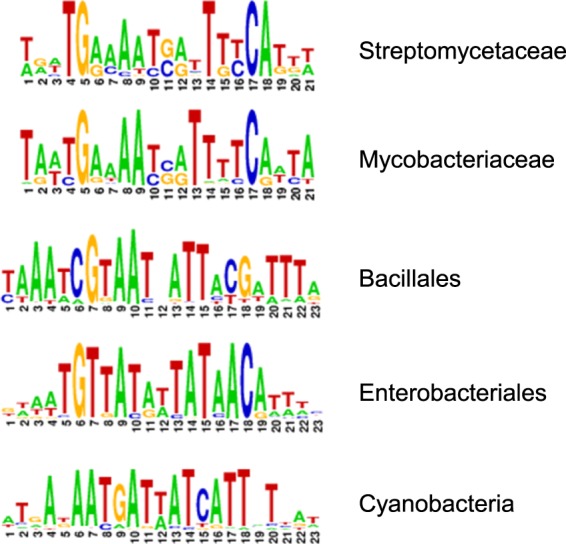
Examples for computationally assembled Zur boxes. The sequence logos are taken directly from RegPrecise, using manually curated regulons [52]. While certain commonalities are evident for the Zur boxes from actinobacteria (Streptomycetaceae and Mycobacteriaceae) and Bacillales, the Zur boxes for enterobacteria (including *E. coli* and *Salmonella*) and cyanobacteria are clearly different.

Residues involved in DNA binding highlighted in cyan in Figure 5C are well conserved between species, including several residues interacting with the phosphate and sugar backbone, and Arg65 which forms hydrogen bonds to N7 of a guanine or adenine base, and thus provides sequence-specific recognition. Since the overall fold of the DNA-binding domains is predicted to be also well conserved, it is likely that these residues also participate in DNA binding in Zur proteins from other species. However, Tyr45, the second residue that directly interacts with a nucleobase, is only present in enterobacterial Zurs and that of *Paracoccus denitrificans*. Therefore, it is reasonable to expect that the recognised DNA sequences differ between species, and this is indeed the case (Figure 7).

The Zur box for EcZur is illustrated in Figure 6B. EcZur binds to the *znuABC* promoter as a dimer of dimers, which is stabilised by a pair of salt bridges between Asp49 and Arg52. These residues are only conserved in enterobacterial and actinobacterial Zurs (Figure 5C). Therefore, the oligomerisation state on DNA is unlikely to be transferrable to other Zurs. Zur boxes for other genomes have been defined from promoter analyses of Zur-regulated genes, and are typically presented as palindromic sequences, for example, 9-1-9 [41], 7-1-7 [53–55], or as 23-bp palindromes [56]. Figure 6B exemplifies that the at least pseudo-palindromic nature of Zur boxes relates to the dimeric nature of the DNA-binding Zur species. It is worth noting that many other metal-sensing transcription factors also bind as dimers, with their cognate DNA sequences also being at least pseudo-palindromes [18–24,57]. Once one or more Zur boxes have been identified for a particular bacterial phylum, genomes can be computationally screened for further putative Zur-binding sites. Definition of a specific motif (TRWNAYRWTATAWYRTNWCA) allowed finding a new Zur-regulated gene in *E. coli*, *pliG* [17]. The manually curated database RegPrecise contains a large collection of Zur boxes and Zur-regulated genes [52].

## Biophysical and biochemical studies on Zur proteins

A range of Zur proteins from different bacteria have been expressed recombinantly and studied in some detail. Table 1 summarises the two most fundamental parameters, namely oligomerisation state and Zn : protein stoichiometry. Since the methods used for protein production may affect these parameters, these are also given.

In most cases, dimers were isolated, but the production of homogeneously metallated Zur proteins is non-trivial. Considering that the structural work discussed above has demonstrated that different Zur proteins can harbour a different number of zinc-binding sites with different functions, it is clear that the determination of accurate metal : protein stoichiometries is the first crucial step towards the understanding of the mechanism of action of any Zur protein. An important common feature is that even in the many instances where the proteins were treated with the metal chelator EDTA [2,2′,2″,2″′-(ethane-1,2-diyldinitrilo)tetraacetic acid], the purified proteins still contained about one zinc ion per monomer. The EDTA-resistant Zn^2+^ is bound to the structural Cys_4_ site. It has remained unclear whether this behaviour is attributable to the site binding zinc with extremely high thermodynamic stability. Given the high affinity (*K*_D_ (Zn-EDTA) = 2.3×10^−14 ^M at pH 7.4 [58]) and the large excesses in which EDTA is typically administered, the Zn^2+^ in the structural site would need to be bound with an affinity well below sub-femtomolar, which is unparalleled in any other Cys_4_ sites in, e.g. zinc fingers or metallothioneins. An alternative explanation may be that the site is kinetically inert. Given that the site is not deeply buried, accessibility is unlikely to be sterically restricted, but it is possible that interaction with EDTA is electrostatically disfavoured.

In those cases where attempts were made to establish the number of total binding sites, stoichiometries of either two or three per monomer were found. In this context, the stoichiometries reported for MtZur and EcZur are particularly instructive. Initial work on MtZur [39] identified only two Zn per monomer, even though three sites were found in the crystal structure. Such discrepancies can have many causes: firstly, metallation during expression may be incomplete or excessive. This can also be a consequence of the chosen tag [59]. Secondly, zinc may be lost during protein purification. Many of the protocols in Table 1 utilise metal-chelating agents including EDTA, DTT, and imidazole. Thirdly, the protein or metal concentrations might not have been determined with sufficient accuracy. In one instance, careful analysis revealed that the conventionally calibrated Bradford assay overestimated EcZur protein concentration by a factor of 2.33 [43]. Finally, it may also be argued that not all metal-binding sites found by X-ray crystallography are necessarily physiologically relevant, but at least in the case of ScZur, the participation of both sites 2 and 3 in zinc sensing has been confirmed (see below) [38]. In contrast, based on stoichiometry data, site 3 in BsZur is believed to be not involved in zinc sensing, even though a site 3 mutant had decreased DNA-binding affinity [12]. The latter was suggested to be due to a decrease in dimerisation tendency of this mutant. Stoichiometric data for enterobacterial Zurs are also not clear-cut: the X-ray structure of *E. coli* Zur contains two Zn per monomer, even though ICP-MS analysis detected an almost 3 : 1 stoichiometry [17]. Similarly, *Salmonella* Zur also binds up to three Zn per monomer [45]. The possibility of a third Zn binding to the structurally disordered C-terminus has been raised [17].

To understand the mode of action and operating ranges of metalloregulatory transcription factors, two thermodynamic parameters are of fundamental importance: the affinity to the cognate DNA and the affinity to the cognate metal (Table 2). The two most frequently used approaches to determine protein–DNA affinities are electrophoretic mobility shift assays (EMSAs) [17,53,54,62] and fluorescence anisotropy (FA) [40,45,47,61]. Both methods can be employed in titrations, where typically the DNA concentration is held constant, and the protein concentration is varied. Evidently, the metallation state of the sensor protein in these assays is critical. The perhaps best-controlled approach employs purified protein with clearly defined metal : protein stoichiometry and buffers that avoid loss of metal. A frequently used alternative approach is to work in EDTA-containing buffers, but supplying an excess of Zn^2+^ [or other metal ion(s)] to ensure saturation of the sensory site(s).

**Table 2:** Affinity of Zur proteins for DNA and Zn^2+^ The DNA-binding affinities refer to fully metallated Zur, except for mutant proteins.

Organism	*K*_D_ (DNA)	*K*_D_ (Zn)
*Bacillus subtilis*	4.3 nM (FA [40]) 6 nM (EMSA [41]) WT: 12.5–25.4 nM, site 2 mutant: 16.5^ ^nM for PrpsNB;* 80.6 nM for PyciC; site 3 mutant: ∼100 nM (EMSA [12])	*K*_D1 _= 5.5 × 10^−14 ^M (0.9 Zn/monomer) *K*_D2 _= 1.2 × 10^−12 ^M (0.5 Zn/monomer) (titration against Quin-2^†^ [40]) *K*_D1 _= 4.2 × 10^−15 ^M (0.1 μM BsZur) = 5.9 × 10^−16 ^M (1.0 μM BsZur) (*In vitro* Zn^2+^ activation assay [40])
*Streptomyces coelicolor*	17.7 nM PrpmG2; 17.6 nM PSCO7682; 74.9 nM PznuA; 68.3 nM PrpmF2 (EMSA [38]) 15 nM PznuA1, 19 nM PznuA2 and PzitB (EMSA [55])	7.8–4.5 × 10^−16 ^M (EMSA, Zn titration in the presence of TPEN [38])
*E. coli*	8.2 ± 0.7 × 10^−18 ^M^2^ (PznuC) 0.053 ± 0.01 × 10^−18 ^M^2^ (PzinT) 0.025 ± 0.01 × 10^−18 ^M^2^ (Pl31p) 520 ± 90 × 10^−18 ^M^2^ (PpliG) (Zur_2_Zn_4_)_2_-Pxxxx; EMSA [17]	9.6 ± 3.0 × 10^−17 ^M (*in vitro* DNA binding) 2.0 ± 0.1 × 10^−16 ^M (transcription assay PznuC [4])
*Salmonella enterica*	Zur_2_Zn_6_: 54 ± 18 nM Zur_2_Zn_4_: 41 ± 10 nM Zur_2_Zn_2_: ≥2.7 ± 0.4 × 10^−5 ^M (FA [45])	*K*_1–2_ 6.36 ± 0.41 × 10^−13 ^M (titration against Quin-2) *K*_3_ 8.04 ± 2.92 × 10^−11 ^M (titration against Quin-2) *K*_4_ ≥5 × 10^−7 ^M (titration against MagFura2^‡^) Indices refer to binding sites on dimer, with structural sites already occupied [44]
*Anabaena* sp. *PCC 7120*	220 ± 10 nM (EMSA [48]) Pall4725: 2.5 nM Pall4723: 7 nM (EMSA [60])	Isothermal titration calorimetry (ITC): [48] ZurZn + Zn ↔ ZurZn_2_, *K*_D_ ∼3.5 × 10^−7 ^M ITC in the presence of DTT: two sites; *K*_D1_ = 6.5 × 10^−7 ^M
*Synechocystis* sp. *PCC 6803*	*K*_D _≤ 55 nM (FA [47,61])	*K_D_*_1_ = 2.3 ± 1.9 × 10^−13 ^M (titration against Quin-2 [47,61])
*Paracoccus denitrificans*	n.d.	*K*_D1_ = 4.0 ± 0.4 × 10^−8 ^M (titration against MagFura2) *K*_D2_ > 1×10^−6 ^M [46]

* Pxxxx: Promoter for gene xxxx. ^†^Quin-2: 2-[(2-amino-5-methylphenoxy)methyl]-6-methoxy-8-aminoquinoline-*N*,*N*,*N*′,*N*′-tetraacetic acid. ^‡^MagFura2: 2-[6-[bis(carboxymethyl)amino]-5-(carboxymethoxy)-2-benzofuranyl]-5-oxazolecarboxylic acid.

The detected species in EMSAs is the DNA, in both its free and protein-bound forms. The DNA employed may be of different length (typically 25–450 bp) depending on how well-defined the location and size of the Zur-box is. For EMSAs to yield unambiguous affinity data, the gels should contain exactly two bands, one for the free DNA, and one for the bound DNA, which in the case of dimeric transcription factors should in the most straightforward cases refer to the 1 : 1 Dimer : DNA complex. In practice, the raw data are often more complicated and may include bands with bound monomer as well as bands with more than one dimer bound, and in more unfavourable cases also diffuse intensity not clearly attributable to either form. The latter effect is due to the complex(es) present in the gel wells dissociating while moving through the gel; this is particularly likely when binding is weak and kinetically labile. FA measurements are a true equilibrium method, yielding data that do not show such complexity and problems. FA may require more stringent experimental design: the detection of protein binding is based on the molecular tumbling rate of fluorescently labelled DNA, which is affected by whether or not a protein is bound. To maximise this effect, the oligonucleotides employed in FA are typically quite short (e.g. 24 bp); hence, the Zur-box sequence needs to be well defined. Both EMSA and FA allow the determination of binding stoichiometry. In the case of EMSA, this can be deduced from measuring the mass of the complex(es) from their in-gel mobility (see, e.g. [17]), while FA titrations at high DNA concentration (approximately two orders of magnitude above *K*_D_) provide the saturation stoichiometry directly. Both data types require fitting to one or more models. The data given in Table 2 refer in most cases to simple 1 : 1 Dimer : DNA binding models, except for the EcZur data which refer to the binding of two dimers per DNA, i.e. the 2 : 1 complexes. All reported dissociation constants for 1 : 1 complexes are in the low to mid-nanomolar range. Recent work has revealed that different promoters within the same genome can have different affinities (see, e.g. the entries for *B. subtilis*, *S. coelicolor*, and *E. coli*); this allows a graded response to different degrees of zinc limitation [17,38,55]. A direct correlation between *in vivo* -fold repression and *K*_D_ was proposed for *E. coli* [17]. In cases of single-dimer binding, *K*_D_s vary over less than two orders of magnitude; in the case of the dimer-of-dimer-binding mode for *E. coli* Zur, the strongest and weakest sites differ by four orders of magnitude — but it should be noted that the units of these constants differ, and therefore values and factors are not directly comparable.

There are several cases where promoters contain more than one Zur box; for example, the *znuA* promoter of *S. coelicolor* [38] and the *yciC* promoter of *B. subtilis* [41] contain at least two Zur boxes, one overlapping with the −35 motif and one with the −10 motif. The significance of this has remained unclear; it is most likely that the two binding sites operate independently, and that this allows for more stringent repression, with the downstream box providing backup in the rare case that repression by the upstream box was not effective [41].

Various methods are available to determine metal–protein affinities. The most direct approach involves mixtures of the protein of interest and a metallochromic dye, into which small aliquots of Zn^2+^ are titrated. The concentration of either free or zinc-bound dye is the measured quantity. Importantly, the dye must have an affinity in a similar order of magnitude as the protein. Otherwise, the two metal-binding molecules cannot compete, and the respective ‘equilibrium constants’ become meaningless.

Activation assays involve studying the DNA-binding ability at different free Zn^2+^ concentrations; this can be monitored *in vitro* by EMSA, or by measuring *in vivo* transcription via a reporter gene under the control of a promoter containing the Zur box. Free [Zn^2+^] is controlled by the addition of well-established zinc chelators such as TPEN [*N*,*N*,*N*′,*N*′-Tetrakis(2-pyridinylmethyl)-1,2-ethanediamine] or EDTA.

The data compiled in Table 2 reflect in most cases the very high affinity of zinc sensor proteins for their cognate metal ion, which is necessary, as intracellular free Zn^2+^ concentrations in bacteria are picomolar or lower [63]. Accordingly, most Zn–Zur dissociation constants are at least in the picomolar range, with two notable exceptions, namely those for *P. denitrificans* and *Anabaena* PCC 7120 Zur, which are likely erroneous due to inappropriate experimental design. In the first case, the detected ‘high’ affinity site — for which *K*_D_ = 40 nM is still at least four orders of magnitude higher than expected — was suggested to refer to binding of Zn to what was thought to be the apo-protein, which had been generated by extensive dialysis [46]. A second site with micromolar *K*_D_ was also found. Considering that all known zinc-binding residues are conserved between *E. coli* and *P. denitrificans* Zur (Figure 5C), the discrepancies between the data for those two proteins are astonishing. It is also surprising that the structural site in *P. denitrificans* Zur apparently remains intact during initial treatment with EDTA (Table 1), but dissociates during dialysis. We note that this report [46] does not mention whether care was taken to prevent protein oxidation during purification, while most other studies were conducted either in the presence of reducing agents [DTT or TCEP; Tris(2-carboxyethyl)phosphine] or under an inert atmosphere. In the case of *Anabaena* PCC 7120 Zur, ITC experiments were conducted both in the presence and absence of DTT. While the presence of DTT enhanced affinity by about one order of magnitude, the *K*_D_s all lie within the micromolar range. This range is what is achievable with the reported experimental design: the ITC experiments involved 20 μM protein, but employing micromolar concentrations of protein (and metal) without a competing agent only allows the determination of *K*_D_s within the same concentration range (± maximally two orders of magnitude), while binding events with higher affinity cannot be quantified.

For the remaining zinc affinity data, there is a clear trend for the data obtained by *in vitro* DNA binding and *in vitro* or *in vivo* transcription assays reporting significantly higher affinities than data obtained by direct titrations of the purified protein in the absence of DNA. Since metal- and DNA-binding equilibria are coupled allosterically, it follows that not only does Zn^2+^ bound to the sensory site increase the affinity of Zur to DNA, but also that DNA bound to Zur will enhance the affinity of the sensory site to Zn^2+^. Such allosteric coupling in metalloregulatory proteins has been quantified and discussed comprehensively by Giedroc and co-workers [64].

EMSA experiments, in conjunction with biophysical characterisation of mutant Zur proteins, have also been instrumental in determining the role of the various Zn-binding sites. When one of the cysteine residues of the structural Zn-binding sites (site 1) was mutated to serine, Zur from both *S. coelicolor* (C90S) [38] and *E. coli* (C103S) [17] was unable to form dimers and bind DNA. CD spectroscopy showed drastic changes in the secondary structure of the C90S mutant ScZur, while mutations affecting sites 2 or 3 did not affect the secondary structure or oligomerisation state [38]. The essential role of the structural site to maintain both the tertiary and quaternary structure is evident. The mutation C88S affecting the sensory site in EcZur completely abolished Zn-responsive transcriptional regulation [17], and the site 2 C79S mutation in ScZur virtually abolished binding to all promoters [38], testifying to the essentiality of this site for zinc sensing.

EMSA experiments have also been employed to explore the metal selectivity of Zur proteins. Typically, this involves removal of the sensory metal(s) by EDTA and supplementing the reaction mixtures with different metal dications (e.g. Zn^2+^, Cd^2+^, Co^2+^, Cu^2+^, Fe^2+^, Mn^2+^, and Ni^2+^). Selectivity is established by assessing whether the supplemented metal can trigger the formation of the DNA–Zur complex. The Zur proteins from *P. denitrificans* [46], *Neisseria meningitidis* [65], and *Brucella abortus* [66] only showed significant complex formation in the presence of Zn^2+^. In contrast, Zur from *Corynebacterium glutamicum* [67] formed Zur–DNA complexes in the presence of Mn in addition to Zn, while Zur from *M. tuberculosis* [68] showed Zur–DNA binding in the presence of Zn, Mn, and Cd. It must be appreciated that sensor proteins do not need to be 100% specific, because *in vivo* not only allostery or stability constants define the sensed metal, but also the cytosolic free metal concentrations [61]. These differ dramatically for different metal ions, and indeed, the Robinson laboratory has found a close correlation between metal sensor *K*_D_s and cytosolic-free concentrations [45,61,63]. Thus, since the described *in vitro* experiments do not emulate the intracellular environment and concentrations of metal and proteins, they may not necessarily reflect likely interactions *in vivo.* It should be added that at least in *S. coelicolor*, Zur is, with a cellular concentration of 3.7 μM, a fairly abundant protein [55].

## Zur regulons

After the discovery of Zur proteins as sensors that regulate the expression of high-affinity zinc uptake systems, many studies have elucidated the full complement of Zur-regulated genes in a range of bacteria (Table 3). Searches for Zur-regulated genes often, but not always, involve the generation of *zur* knockout mutants. This also enables the determination of phenotypes. Δ*zur* mutants often have higher zinc contents, and suffer from increased sensitivity towards elevated concentrations of zinc, although there are exceptions to that rule, for example, in *Corynebacterium diphtheriae* [73]. The viability and/or virulence of Δ*zur* mutants of pathogenic bacteria in the host is often more or less severely compromised [74,85,86,95], but that is not always the case [75]. The knockout phenotypes highlight that Zur provides tolerance towards both high and low zinc: it is required not only to boost zinc uptake under limiting conditions but also to down-regulate zinc uptake in excess conditions.

**Table 3:** Genes of the Zur regulons from a range of bacteria

	Species	Experimental approaches	Zn import	COG0523	Ribosomal proteins	Enzymes	Other	Activation	Refs.
Actinobacteria	Streptomyces coelicolor*	Δ*zur* mutant; qRT-PCR. Forty-one putative Zur boxes identified by CHiP [55]	***znuABC***	***yciC***	***rpmE2***, ***rpmF2***, ***rpmB2***, ***rpmG2***, ***rpsN2***, ***rpsR2***	***SCO7676*** (***Putative fer-redoxin***), ***7681***, ***7682*** (Coelibactin biosynthesis)	***SCO0472***, ***0474***, ***3431*** (various)	***zitB*** (CDF effluxer)	[16,55,69,70]
	*Mycobacterium tuberculosis*	Δ*zur* mutant, microarrays, qRT-PCR. Thirty-two genes (probably 16 operons) up-regulated	***znuABC***	***yciC***	***rpmB1 and B2***, ***rpmG1***, ***rpsN2***, ***rpsR1***		Secretory proteins involved in virulence		[68]
	*Mycobacterium avium* ssp. *paratuberculosis*	Exposure to metal starvation, qRT-PCR, RNAseq. Zinc-responsive genomic islands (ZnGI)	***mptABC***, ***mptDEF***	3× ***cobW***	***rpsR2***, ***rpsN2***, ***rpmG2***, ***rpmE2*** *rpmB1*, *rpmG1*	***sidAB* and *G***; metallophore bio-synthesis and export	Secretory proteins involved in virulence; *lamB*		[71]
	*Corynebacterium glutamicum*	Δ*zur* mutant, microarrays, qRT-PCR; nine genes differentially regulated in mutant	2× ***znuABC***	***cg0794*** *(**yciC**)*		***cg0795*** (*oxidoreductase*) *cg3107* (*adhA*)	***cg0040*** *(secreted protein)*	***zrf*** (CDF effluxer) ***zra*** (ATPase)	[67,72]
	*Corynebacterium diphtheriae*	ΔZur mutant, qRT-PCR, reporter assays	***troABC***	***zrg** (**yciC**)*		*adhA*	*Several surface-anchored proteins*		[73]
Firmicutes	*Bacillus subtilis*	Δ*zur* mutant, microarrays, quantitative RT-PCR	***znuABC*** (annotated as *adcABC*), ***ZinT***	***yciC***	***rpsN2***, ***rpmE2***	***folE2*** (***yciA***), ***yciB*** *ytiB* (carbonic anhydrase)			[12,53]
	Listeria monocytogenes*	Δ*zur* mutant, quantitative RT-PCR	***znuABC*** (2×)		*rpsN2*				[74]
	Staphylococcus aureus*	Transcription assays	***adcBC***, *adcA-II* *zur*	*yciC*	*rpsN2*, *rpmG2*		* *		[75]
	Enterococcus faecalis*	Exposure of WT to high [Zn]; microarrays, qPCR	***adcBC***, ***adcA-II***, ***adcA***	*yciC*	*rpmF2* (*x2*), *rpsN2* (*x2*), *rpmG2* (*x2*)		* *		[76]
	*Streptococcus suis*	Δ*zur* mutant, microarrays, 121 genes (72 up-, 49 down-regulated in mutant); qRT-PCR	None of the commonly Zur-regulated genes. Several enzymes and membrane proteins up-regulated in mutant, e.g. Zn-dep. NADPH-quinone reductase and 3-phosphatidyltransferase. Unclear whether genes have Zur boxes in the upstream region. Alternative sensor AdcR more common in Streptococci.						[42]
Gamma-proteobacteria	*E. coli*	Δ*zur* mutant, qRT-PCR, EMSA	***znuABC***, ***zinT***		***rpmE2**,* *rpmJ2*	***pliG***			[17,77]
	Pseudomonas aeruginosa*	Δ*zur* mutant, qRT-PCR; WT grown in high and low Zn, microarray, qRT-PCR; RNASeq of ΔznuA mutant	***znuABC***, ***znuD*** (***TonB-dR***), ***zrmABCD*** (***tonB-dR***)	***yciC***	***rpmE2**,* ***rpmJ***	***folE2***, ***amiA***, ***can***, ***pyrC2***, ***PA5537*** (glutamine synthetase) ***cntOLMI*** (metallophore synthesis)	***dksA***, *zbp*	*** ***	[78–81]
	Pseudomonas protegens*	Exposure to Zn limitation; microarrays (73 genes up-, 28 down-regulated); qRT-PCR	***znuABC*** (*3×*), ***3****× **tonB-dR***	**yciC**, **yciC2**	***rpmJ**, **rpmE1***	***folE2***, ***amiC***, ***can***, ***pyrC2***, ***hisI2***	***dksA***		[82]
	*Yersinia pestis*	Δ*zur* mutant, microarrays; 154 differentially regulated genes in response to high [Zn]	***znuABC*** (*2×*)		***rpmJ2*** *rpmE2*				[83]
	*Vibrio cholerae*	Bioinformatics and biochemical promoter analysis	***znuABC***, ***zrgABC***(***DE***)		*rpmE2*, *rpmJ2*	*ribA*	***zrgD*** and ***E***: hypothetical proteins		[84]
	Acinetobacter baumannii*	Δ*zur* mutant, 76 genes up- and 68 genes down-regulated, qRT-PCR	***znuABC***, ***3****× **tonB-dR***, ***ExbD***, ***ExbB***	***zigA***	***rpmE2***				[85]
	*Xanthomonas campestris*	Δ*zur* mutant, microarrays, 64 putative Zur-regulated ORFs; *in vitro* transcription assays	***znuABC*** *2× tonB-dR*	***XC0267***		*folE2*, *amiC*	***hrpX***; involved in pathogenicity	***czcD*** (CDF effluxer)	[86,87]
	*Francisella tularensis and F. novicida*	Δ*zur* mutant, RNASeq, qRT-PCR	***zupT*** (***FN***)	***FTN_0880***			***FTN_0395;*** ArsR family transcrip-tional regulator		[88]
Alpha-proteobacteria	*Caulobacter crescentus*	Microarrays, transcription assays. Twenty-eight genes (7 up-, 21 down-) regulated in Δ*zur* mutant	***znuGHI*** **3× *tonB*-dR** (***znuK***, ***L*** and ***M***)	***zrpX***	*rpUI*, *rpmA*		***zrpW*** (putative transporter)	**RND systems**, **ATPase**, ***tonB-dR*s**	[89]
	Agrobacterium tumefaciens*	Δ*zur* mutant, qRT-PCR	***znuABC/troABC***, ***zinT***	***yciC*** (2×)					[90,91]
	Paracoccus denitrificans*	RNASeq (Zn-chelated/depleted/replete), 147 genes (133 up-, 14 down-regulated in low [Zn]), qRT-PCR	***znuABC***, ***aztABC***	***yciC***					[46]
Beta-proteobacteria	*Neisseria meningitidis*	Δ*zur* mutant, microarrays, qRT-PCR; 17 genes differentially regulated in mutant	***znuABC***, **2× *TonB-dR***		***rpmE2**, **rpmJ***	***queC**, **queF***		***adhP***	[65]
	*Cupriavidus metallidurans*	Δ*zur* mutant, microarrays, Zur binding to promoter regions was tested	***zupT*** *tonB-dR*	**Op0317f** ***cobW*1 *W2***, ***W3***	No	***dehH2***	***dksA1***	σ-factor ***fliA***; ***cadA*** but not ***zntA***	[92,93]
Cyanobacteria	*Anabaena PCC 7120*	Promoter mapping, screening for putative Zur boxes, qRT-PCR, 23 genes identified [60] Δ*zur* mutant, semiquant. qRT-PCR, EMSA [62]	***znuABC***, ***3 tonB-dR***, (***alr3243***, ***alr4031***)	***alr1197***, ***all1751***, *all4722*		***hemB2***, ***thrS2***, ***folE2***, **glycosyl-transferase** [60]; ***sodA***, **catalase**, **peroxiredoxin** [62], several more predicted	***aztR*** ***all1474**, **alr3495***		[60,62]
	Synechococcus PCC 7002*	Δ*zur* mutant, RNASeq	***znuABC***			***hemB2***, ***folE2*** (+2 more)			[94]

In most cases, Zur regulation has been confirmed experimentally. Some entries have been complemented by data extracted from the RegPrecise database [52]; experimentally confirmed Zur-regulated genes are printed in bold. Actinobacteria and Firmicutes have a single membrane; all other bacterial groups are Gram-negative and have an outer and inner membrane and a periplasm. Asterisks (*) indicate species in which *zur* expression is subject to autoregulation. Also see Figure 1 regarding uptake/efflux proteins. Abbreviations: *znuABC*, *znuGHI*, *troABC*, *adcABC*, *aztABC*, *zrgABC* are all ABC-type zinc uptake systems; *zinT* and *adcA*: periplasmic zinc-binding proteins; the latter has both a ZnuA and ZinT-like domain; *zupT*: zinc importer of the ZIP family; *zitB*: zinc exporter of the cation diffusion facilitator (CDF) family; *tonB-dR*: TonB-dependent receptor; *exbB/D*: parts of energy transduction system for TonB-dependent receptors; *oprD*: outer-membrane porin; *aztR*, *smtB*: zinc excess sensors; *cobW*/*yciC*: frequently used labels for COG0523 proteins; ribosomal proteins: *rpmE *= L31, *rpmJ *= L36, *rpUI *= L21, *rpmA *= L27, *rpmB *= L28, *rpmG *= L33, *rpsN2 *= S14p, *rpsR1 *= S18, *rpmF2 *= L32p; *zrpW*: zinc-regulated protein; *zbp*: putative zinc-binding protein; *pliG*: periplasmic lysozyme inhibitor; *dksA*: zinc-independent transcription factor; *can*: gamma-carbonic anhydrase; *pyrC2*: dihydroorotase; *amiA*/*amiC*: *N*-acetyl-muramoyl-l-alanine amidase; *adhA*: zinc-dependent alcohol dehydrogenase; *adhP*: alcohol dehydrogenase; *folE2* and *ribA*: GTP cyclohydrolases; (tetrahydrofolate biosynthesis); *dehH2*: haloacetate dehydrogenase; *hemB*: delta-aminolevulinic acid dehydratase (tetrapyrrole biosynthesis); *thrS2*: threonyl-tRNA synthetase; *hrpX*: hypersensitivity-pathogenicity regulatory gene; *queC*: 7-cyano-7-deazaguanine synthase; *queF*: NADPH-dependent 7-cyano-7-deazaguanine reductase; Other abbreviations such as alr1197 and XC0267 are locus tags.

To determine Zur regulons, wild-type and mutant strains are subjected to low and high zinc concentrations, and the expression of either all or a limited number of pre-selected genes is assessed, typically by microarrays or quantitative reverse-transcriptase polymerase chain reaction (qRT-PCR), under these four conditions. A gene that is Zur-regulated is expected to be regulated by zinc concentration in the wild-type, but not in the Δ*zur* strain. This is a necessary but not sufficient criterion, because the consequences of deleting *zur* may be more complex: (i) Zur may regulate the expression of other transcription factors (including other urs, SmtB-like excess sensors, and sigma factors; see Table 3), or be itself regulated by other transcription factors. (ii) The deletion of *zur* may lead to general disturbance of metal homeostasis, with potential indirect consequences, including effects on other metal sensors and on the cellular redox balance. Although zinc is not itself redox-active like copper or iron, its levels may affect redox balance through its frequent association with redox-active thiols [96], and therefore, concurrence of oxidative stress protection and zinc homeostasis is expected. Indeed, higher zinc levels protect *B. subtilis* from increased H_2_O_2_ concentrations [53]. Examples of indirect effects on gene transcription in knockout or overexpressing Zur mutants include potential mis-metallation of Fur at high [Zn^2+^] in a Δ*zur* mutant causing repression of Fur-regulated genes in *Caulobacter crescentus* [89] and interplay between zinc homeostasis and disulfide stress via Zur and sigma factor R in *S. coelicolor* [69]. Furthermore, the pleiotropic phenotype of Δ*zur* and *zur*-overexpressing mutants, extensive cross-talk between Zur, Fur, and PerR, and up-regulation of antioxidant enzymes (superoxide dismutase sodA, catalase alr0998, and peroxiredoxin gct3) in a Δ*zur* mutant of the cyanobacterium *Anabaena* sp. PCC 7120 probably also include both direct and indirect effects [48,62,97,98]. In turn, the levels of metal ions other than Zn^2+^ may also affect the expression of members of Zur regulons, as shown for *Enterococcus faecalis* [76], where exposure to high levels of Cu^2+^ activates Zur-regulated zinc uptake, presumably to protect from mis-metallation of Zn-requiring proteins and from oxidative stress.

The identification of a true member of a Zur regulon therefore also requires: (i) the presence of Zur boxes, (ii) proof that Zur binds to the promoter region of the selected gene, and/or (iii) *in vivo* reporter assays. A recent study illustrates these requirements dramatically: Of the hundreds of genes that were differentially expressed in a Δ*zur* mutant of *Cupriavidus metallidurans*, only 11 had computationally identified Zur boxes, and of those, only four were experimentally verified to be true Zur boxes [92].

In many bacterial species, besides repressing uptake systems, Zur can also repress genes encoding paralogues of ribosomal proteins. These paralogues generally constitute non-Zn-requiring alternatives of ribosomal proteins that require Zn^2+^ for folding. The expression of zinc-free alternatives under Zn-depletion conditions liberates Zn^2+^ from ribosomes, which is then available for other cellular processes in which Zn^2+^ is indispensable. Since ribosomes are highly abundant in rapidly growing cells, the presence of two or three zinc-containing ribosomal proteins represents a large reservoir of zinc, and this may, in fact, account for the majority of intracellular zinc [16,70]. This was first discovered for *B. subtilis* [99], but it is now evident that many bacteria utilise this strategy (see Table 3).

Zur-regulated expression of enzymes is also widespread. In many cases, these enzymes are zinc-free paralogues of otherwise zinc-requiring enzymes, for example, alcohol dehydrogenase and delta-aminolevulinic acid dehydratase. These alternatives are thought to decrease the cell's requirements for zinc. A further common occurrence are putative small GTPases of the COG0523 family [100]. These are frequently annotated as *yciC*, the label for this gene in *B. subtilis*. Other members of this family are involved in the maturation of metal-requiring enzymes (e.g. Fe-requiring nitrile hydratase) and cofactors (e.g CobW for cobalamin biosynthesis; note that mis-annotation of zinc-related COG0523 members as *cobW* is frequent), which has led to the suggestion that these proteins may function as metallochaperones. Precisely how they might contribute to zinc homeostasis has remained unclear, although their zinc-binding ability and consequences thereof have been demonstrated [101].

In several actinomycetes and some pathogenic bacteria, biosynthetic clusters for the synthesis of metallophores are under the control of Zur, for example, coelibactin in *S. coelicolor* [70], ethylenediamine-disuccinate in *Amycolatopsis japonicum* [54], and pseudopaline in *Pseudomonas aeruginosa* [78,79]. Typically, such metallophores are secreted to capture scarce zinc, and the resulting complexes are then taken up by TonB-dependent receptors which mediate active transport through the outer membrane of Gram-negative bacteria. In *P. aeruginosa*, both pseudopaline and TonB-dependent receptor production are under the control of Zur, and there are now many more examples of Zur-regulated TonB-dependent receptors (Table 3). These receptors and the metallophores they transport play a crucial role in zinc acquisition by pathogenic bacteria [5].

In several genomes (e.g. *S. coelicolor*, *Pseudomonads*, and *P. denitrificans*), Zur is subject to autoregulation, i.e. represses its own transcription in zinc-replete conditions. This only seems to be the case when the *zur* gene is part of an operon, e.g. with *znuABC* [56]. Conversely, the expression of Zur in Mycobacteria [39] and *C. diphtheriae* [73] is inducible by high [Zn^2+^]. In these species, *zur* is co-transcribed with the gene for an SmtB-like zinc excess sensor.

## Beyond repression: Zur as a transcriptional activator

Evidence has been emerging that in at least some bacteria, Zur proteins may also activate the transcription of genes. Two of the best-studied cases concern Zur-regulated expression of metal efflux proteins of the CDF family in *Xanthomonas campestris* [86] and *S. coelicolor* [55]. Zur binding to their promoter regions was studied in both cases. In *X. campestris*, a GC-rich 59-bp sequence that shows no significant similarity with Zur boxes in this organism was identified by DNAse footprinting in the upstream region of the gene *xc2976*, which codes for a CDF effluxer. The size of the protected region suggests that more than one dimer is required for binding and activation. In *S. coelicolor*, the promoter region of the *zitB* gene contains a Zur box upstream of the −35 site, with similar affinity to other Zur boxes in this organism (15–20 nM). However, since the *zitB* Zur box does not overlap with the RNA polymerase-binding site, this interaction is not expected to lead to repression. Activation occurs at higher [Zn^2+^], apparently through Zur oligomerisation, as judged from the expansion of the DNA footprint. Higher-order oligomers have recently been detected for several Fur-family proteins [29]. Zinc-dependent activation of gene expression under zinc-rich conditions was also observed in *N. meningitidis* [65] and *C. crescentus* [89]. The expression of two efflux proteins in *C. glutamicum* is also Zur-dependent [72]. Here, a new 10-1-10 direct repeat sequence binds Zur in which sensory sites are not populated. This recognition site overlaps with the −35 site, resulting in repression at low [Zn^2+^]. Thus, it can be anticipated that there is significant variation between species in activation/de-repression mechanisms that deviate from the canonical mode of action.

## Conclusions

Zinc sensors of the Zur family are ubiquitous, present in most Gram-negative and many Gram-positive bacteria, with the number of recognised Zur-regulated genes still increasing. By governing the expression of zinc-supplying and zinc-requiring proteins, they provide regulation of intracellular total and free Zn^2+^ concentrations. This protects bacteria against both high and low external Zn^2+^ concentrations, which is of particular interest regarding pathogens [7] but also human microbiomes [102]. Although protein folds are well conserved between Zur proteins from different bacterial phyla, there is surprising diversity regarding the position of the sensory Zn-binding residues in the protein sequence. Yet in all cases where this is known, the tetrahedral coordination sphere of the (major) sensory site consists of two nitrogens, one oxygen and one sulfur. This seems to be particularly suited to Zn^2+^, providing a very high affinity in the atto- to femtomolar range. Some Zur proteins seem to fine-tune DNA affinity by employing an additional zinc site, which extends the range at which Zn^2+^ can be sensed, and allows for differential expression of several sets of genes. Zur–DNA interactions can also be modulated by employing different degrees of oligomerisation, and the number and type of Zur boxes. Moreover, Zur proteins may also act as activators of transcription, and there even appear to be cases where the allosteric switch has the opposite effect, namely increased DNA affinity in the absence of zinc. In the face of this variety, it is clear that our understanding of structures and dynamics of Zur and its biomolecular complexes is as yet far from comprehensive.

## Abbreviations

EDTA: 2,2′,2″,2″′-(ethane-1,2-diyldinitrilo)tetraacetic acid
EMSAs: electrophoretic mobility shift assays
FA: fluorescence anisotropy
Fur: ferric uptake regulator
qRT-PCR: quantitative reverse-transcriptase polymerase chain reaction
TPEN: *N*,*N*,*N*′,*N*′-Tetrakis(2-pyridinylmethyl)-1,2-ethanediamine
Zur: zinc uptake regulator
